# Transcriptomic analysis reveals the regulatory mechanisms of messenger RNA (mRNA) and long non-coding RNA (lncRNA) in response to waterlogging stress in rye (*Secale cereale* L.)

**DOI:** 10.1186/s12870-024-05234-x

**Published:** 2024-06-12

**Authors:** Daniel Bimpong, Lili Zhao, Mingyang Ran, Xize Zhao, Cuicui Wu, Ziqun Li, Xue Wang, Ling Cheng, Zhengwu Fang, Zanmin Hu, Chengming Fan, Bernard Gyebi-Nimako, Yirou Luo, Shuping Wang, Yingxin Zhang

**Affiliations:** 1 College of Agriculture, Yangtze University, Jingzhou, 434000 Hubei China; 2 State Key Laboratory of Plant Cell and Chromosome Engineering, Institute of Genetics and Developmental Biology, Innovation Academy for Seed Design, Chinese Academy of Sciences, Beijing, 100101 China

**Keywords:** Rye (*Secale cereale* L.), DE-lncRNA, DE-mRNA, Transcriptome sequencing, Waterlogging stress (WS)

## Abstract

**Background:**

Waterlogging stress (WS) negatively impacts crop growth and productivity, making it important to understand crop resistance processes and discover useful WS resistance genes. In this study, rye cultivars and wild rye species were subjected to 12-day WS treatment, and the cultivar *Secale cereale* L. Imperil showed higher tolerance. Whole transcriptome sequencing was performed on this cultivar to identify differentially expressed (DE) messenger RNAs (DE-mRNAs) and long non-coding RNAs (DE-lncRNAs) involved in WS response.

**Results:**

Among the 6 species, *Secale cereale* L. Imperil showed higher tolerance than wild rye species against WS. The cultivar effectively mitigated oxidative stress, and regulated hydrogen peroxide and superoxide anion. A total of 728 DE-mRNAs and 60 DE-lncRNAs were discovered. Among these, 318 DE-mRNAs and 32 DE-lncRNAs were upregulated, and 410 DE-mRNAs and 28 DE-lncRNAs were downregulated. GO enrichment analysis discovered metabolic processes, cellular processes, and single-organism processes as enriched biological processes (BP). For cellular components (CC), the enriched terms were membrane, membrane part, cell, and cell part. Enriched molecular functions (MF) terms were catalytic activity, binding, and transporter activity. LncRNA and mRNA regulatory processes were mainly related to MAPK signaling pathway-plant, plant hormone signal transduction, phenylpropanoid biosynthesis, anthocyanin biosynthesis, glutathione metabolism, ubiquitin-mediated proteolysis, ABC transporter, Cytochrome b6/f complex, secondary metabolite biosynthesis, and carotenoid biosynthesis pathways. The signalling of ethylene-related pathways was not mainly dependent on AP2/ERF and WRKY transcription factors (TF), but on other factors. Photosynthetic activity was active, and carotenoid levels increased in rye under WS. Sphingolipids, the cytochrome b6/f complex, and glutamate are involved in rye WS response. Sucrose transportation was not significantly inhibited, and sucrose breakdown occurs in rye under WS.

**Conclusions:**

This study investigated the expression levels and regulatory functions of mRNAs and lncRNAs in 12-day waterlogged rye seedlings. The findings shed light on the genes that play a significant role in rye ability to withstand WS. The findings from this study will serve as a foundation for further investigations into the mRNA and lncRNA WS responses in rye.

**Supplementary Information:**

The online version contains supplementary material available at 10.1186/s12870-024-05234-x.

## Background

Waterlogging stress (WS) is one of the key abiotic stresses affecting crop productivity due to global climate change [1], particularly in the lower and middle Yangtze River basins of China, where the majority of crops are cultivated. In this region, most farms lack effective drainage systems, resulting in frequent water levels exceeding the crop requirements and leading to annual production losses [2, 3]. Unfortunately, the situation has worsened due to the rise in severe weather conditions triggered by the effects of global warming [4]. Furthermore, the focus on high yield and food quality in plant breeding has led to a loss of genetic diversity and stress-tolerance genes over time [5]. Therefore, it is important to identify WS resistance genes and enhance crop stress tolerance in these regions. During WS, plants experience oxygen deprivation as carbon dioxide and oxygen cannot efficiently transfer between the root zones. WS prevents the exchange of gases between plants and their environment, leading to waterlogged plant parts and restricting the production of energy in the mitochondria through heterotrophic processes [5]. To adapt to low oxygen levels, plants undergo modifications in their physiology, metabolism, and growth. These changes involve significant reprogramming of RNA molecules, including coding RNA (mRNA) and non-coding RNA (ncRNA) [6]. Studies investigating gene transcription changes in response to oxygen deficiency have been conducted on rice [7] and maize [6]. These studies have demonstrated the similarity of gene responses to oxygen deficiency across species [8, 9].

Long non-coding RNAs (lncRNAs) are RNA molecules longer than 200 nucleotides that do not encode proteins but play a role in influencing biological processes [10]. They can affect the regulation of other genes in a *cis* or *trans* manner and various processes such as alternative splicing, translation, and posttranscriptional gene epigenetic regulation [11]. Several lncRNAs have been identified in various plant species, including maize [12], barley [13], rice [14], and wheat [15]. The advent of high-throughput RNA sequencing (RNA-Seq) technology has facilitated the discovery of lncRNAs involved in biotic and abiotic stress responses in plants. LncRNAs have a crucial function as biological regulators in animals and plants, influencing various developmental processes and responding to biotic and abiotic stress. They perform a variety of activities at the transcription, posttranscriptional, and epigenetic levels, and have the ability to target specific stress-responsive mRNAs [16]. Changes in individual mRNA levels in total RNAs are observed, and the translational efficiency of specific mRNAs dynamically alters in response to different abiotic stresses. It is believed that the targeted control of protein production from specific mRNA molecules is essential because these proteins play an important function in how plants respond to environmental conditions [17]. Nonetheless, only a limited number of WS-responsive lncRNAs and mRNAs in plants have been thoroughly characterised, particularly in terms of their regulatory mechanisms. Hypoxia is a common occurrence in waterlogged environments, particularly in crop organs. Research on cassava has highlighted the significance of lncRNAs as regulators of WS [18]. Studies conducted on cucumbers have identified several lncRNAs with regulatory roles in the expression of specific genes under hypoxic stress. For instance, *TCONS_00019494* influences how the ATP-dependent RNA helicase gene is expressed. *TCONS_00015763* acts as a regulator of syntaxin, specifically during the development of tolerance to hypoxic stress. *TCONS_00003967* has the potential to control the expression of the gene encoding xyloglucan endotrans-glucosylase/hydrolase. *TCONS_00008071* targets the malate dehydrogenase gene. *TCONS_00019433* can affect the gene encoding the 26 S proteasome non-ATPase regulatory subunit [19]. Despite these findings, the regulatory functions of lncRNAs and mRNAs in WS stress across various plant species remain limited. Therefore, there is a need for comprehensive identification and characterisation of lncRNAs and mRNAs involved in the regulation of WS in plants.

Rye, a crop cultivated for generations across Europe and Asia, holds the distinction of being the second most important grain, after wheat, used in baking bread and other products. Rye belongs to the Poaceae plant family, specifically the subfamilies Pooideae and Triticeae, along with other grains such as wheat and barley. Most cultivated rye species trace their ancestry back to the perennial grass *Secale montanum*, which can still be found growing in its native habitat of southern Europe [20]. Despite being grown in many countries, rye is considered a minor crop in terms of overall output due to its requirement for colder growth conditions, the wide availability of alternative regional crops for rye-based goods, and the distinct distribution patterns of rye production compared to wheat [20]. Rye is renowned for its exceptional soil adaptability and high tolerance to abiotic stress. To effectively regulate its growth, development, and adaptation to stressful environments at both the physiological and molecular levels, rye has evolved intricate mechanisms [21]. The precise regulatory functions of lncRNAs and mRNAs in response to WS in plants, especially in rye, are not well understood, even though WS is becoming more common and has a major impact on crop productivity. Therefore, it is necessary to comprehensively identify and characterise these genetic elements to enhance rye adaptation to WS, which is crucial for the development of improved stress-tolerant cultivars.

This study aims to identify and characterise the lncRNAs and mRNAs involved in the regulatory processes of WS response in rye. It seeks to provide insights into the key genes and regulatory processes involved in rye adaptation to WS. To achieve this, we examined the expression patterns and regulatory responses of lncRNAs and mRNAs in 12-day rye seedlings subjected to waterlogging treatment. We utilised transcriptome sequencing, classification and enrichment analysis of GO annotations and KEGG pathways, RT-qPCR, co-expression, and protein-protein interaction (PPI) analysis for this study. The insights gained from this research will contribute to the identification of key genes that actively contribute to rye ability to adapt to WS. Furthermore, these findings will provide valuable insights for breeders engaged in rye breeding programmes.

## Results

### Phenotypic and physiological analysis of different rye species under waterlogging treatment

**Fig. 1:**
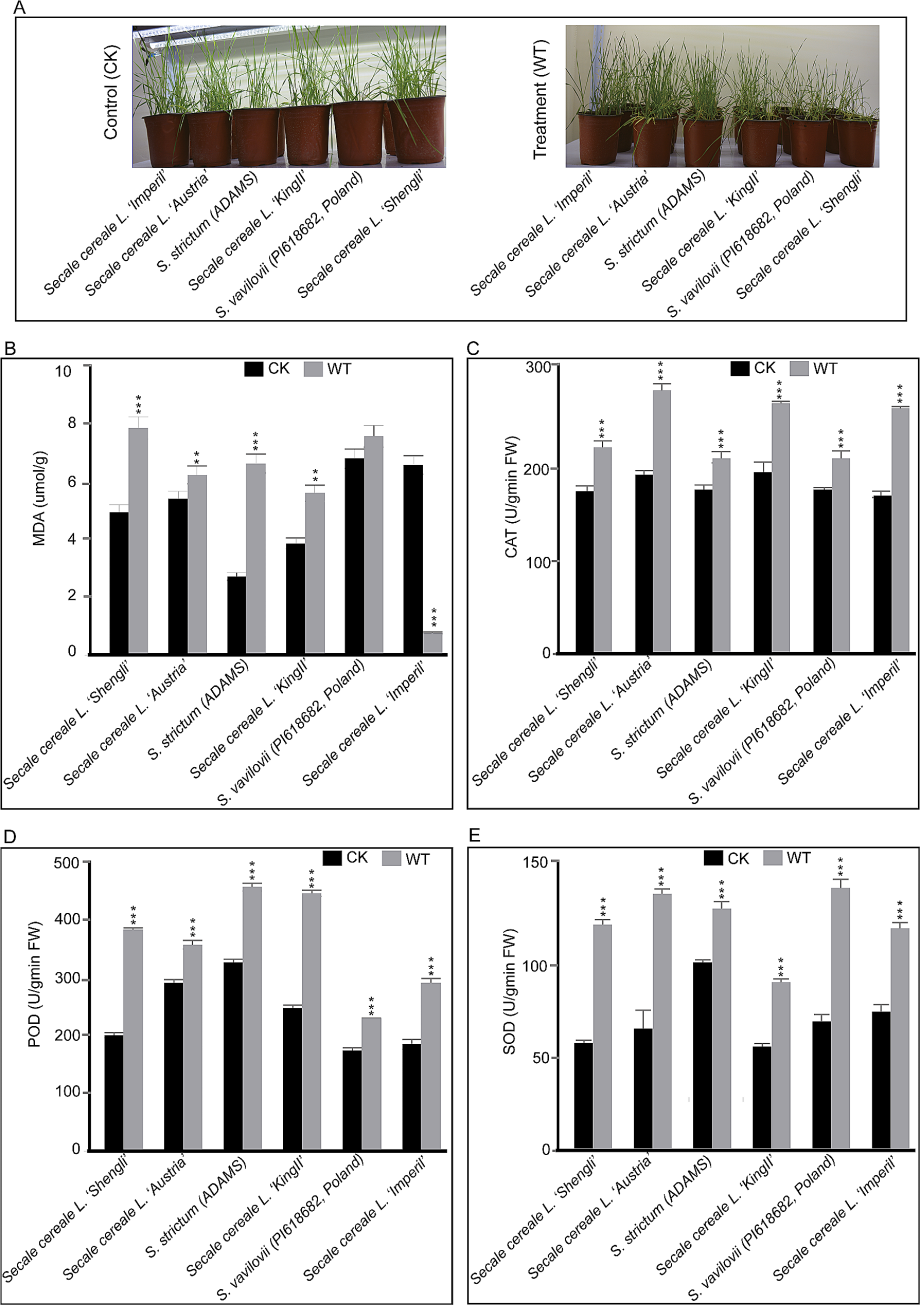
Treatment and physiological parameters. Waterlogging stress (WT) and control (CK) seedlings of four rye cultivars and two wild species (**A**), malondialdehyde content **(B)**, and enzymatic antioxidant activities of catalase **(C)**, peroxidase **(D)**, and superoxide dismutase **(E)** of WT and CK seedlings. Significant differences between three biological replicates are denoted by asterisks, indicating statistical significance at *p* < 0.05. The levels of significance are indicated as *p* < 0.001 (**) and *p* < 0.0005 (***)

To identify rye species with an efficient defence response against WS, treatment was performed on four rye cultivars (*Secale cereale* L. Imperil, *S. cereale* L. Austria, *S. cereale* L. King II, and *S. cereale* L. Shengli) and two wild rye species (*S. strictum* (ADAMS) and *S. vavilovii* (PI618682, Poland). After 12 days of WS, a reduction in shoot height was observed in all the stressed seedlings, especially in *S. vavilovii* and *S*. *cereale* Shengli. Additionally, leaf chlorosis, characterised by yellowing, was observed in the stressed samples. *S*. *cereale* Imperil displayed the mildest symptoms, with no obvious changes observed except for the shoot height. For *S*. *cereale* Austria and *S. strictum*, little seedling wilting was observed, along with the tips of the older leaves turning yellow. Severe symptoms were observed in *S*. *cereale* King II, the whole older leaves turned yellow. The most severe symptoms were observed in *S. vavilovii* and *S*. *cereale* Shengli, not only did their oldest leaves turn yellow, but some became rotten. Some seedlings had difficulty growing under WS conditions, especially *S*. *cereale* Shengli (Fig. 1A).

To analyse the physiological changes caused by WS, the physiological parameters of the treatment and control seedlings were examined. The concentration of malondialdehyde (MDA) serves as an indicator of oxidative damage and lipid peroxidation. In normal growth conditions, the amount of MDA varied among different species. *S*. *cereale* King II had the highest MDA levels, followed by *S*. *cereale* Imperil, *S*. *vavilovii*, *S*. *cereale* Shengli, *S*. *cereale* Austria, and *S*. *strictum*, which had the lowest MDA levels. However, after 12 days of waterlogging treatment, the MDA contents of all the stressed seedlings, except for *S*. *cereale* King II, were significantly different compared to the control groups (Fig. 1B). The MDA content increased by 59.4% in *S*. *cereale* Shengli, 15.6% in *S. vavilovii*, 147.2% in *S. strictum* (ADAMS), 45.7% in *S*. *cereale* Austria, and 11.5% in *S*. *cereale* King II, while there was a decrease of 89.2% in *S*. *cereale* Imperil. SOD activity indicates the seedling’s ability to neutralise harmful superoxide radicals; CAT content reflects the capacity to detoxify hydrogen peroxide; and POD demonstrates their ability to cope with oxidative stress. There was a significant increase in the levels of POD (Fig. 1D), SOD (Fig. 1E), and CAT (Fig. 1C) activities in all the treatment samples compared to the control. In the POD activity, there was an increase of 92.4% *in S*. *cereale* Shengli, 22.9% in *S. vavilovii*, 40.2% in *S. strictum* (ADAMS), 80.7% in *S*. *cereale* Austria, 32.3% in *S*. *cereale* King II, and 58.5% in *S*. *cereale* Imperil. Regarding SOD activity, there was an increase of 112.5% in *S*. *cereale* Shengli, 112.6% in S. vavilovii, 28.9% in *S. strictum* (ADAMS), 62.9% in *S*. *cereale* Austria, 104.6% in *S*. *cereale* King II, and 61.2% in *S*. *cereale* Imperil. Furthermore, for CAT activity, there was an increase of 27.5% in *S*. *cereale* Shengli, 47.3% in *S. vavilovii*, 19.1% in *S. strictum* (ADAMS), 38.1% in *S*. *cereale* Austria, 19.1% in *S*. *cereale* King II, and 55.5% in *S*. *cereale* Imperil. Based on these results, the cultivar *S*. *cereale* Imperil showed higher tolerance to WS (Table 1) and was subsequently selected for whole-transcriptome sequencing.

**Table 1:** The morphological effect of waterlogging on *Secale cereale* L. Imperil roots and leaves

Sample	Root length (cm)	Leaf length (cm)	Fresh leaf weight (g)	Dry leaf weight (g)	Fresh root weight (g)	Dry root weight (g)
CK1	13.76 ± 2.3	30.56 ± 4.1	0.7874 ± 0.2	0.6978 ± 0.17	0.4544 ± 0.19	0.4254 ± 0.12
CK2	14.01 ± 2.8	31.14 ± 3.6	0.7912 ± 0.08	0.6997 ± 0.1	0.4631 ± 0.11	0.4279 ± 0.16
CK3	13.96 ± 2.7	31.56 ± 2.4	0.8013 ± 0.13	0.7105 ± 0.21	0.4810 ± 0.08	0.4321 ± 0.14
WT1	7.71 ± 1.3	25.05 ± 2.1	0.6301 ± 0.2	0.5421 ± 0.16	0.2460 ± 0.06	0.2271 ± 0.08
WT2	7.94 ± 2.6	24.98 ± 2.7	0.5987 ± 0.15	0.5234 ± 0.15	0.2422 ± 0.10	0.2275 ± 0.06
WT3	7.55 ± 1.1	24.62 ± 3.3	0.6145 ± 0.19	0.5387 ± 0.18	0.2452 ± 0.09	0.2345 ± 0.04

### Transcriptome profile of waterlogged stress rye seedlings

In this study, leaves from 12-day waterlogged (WT) *S. cereale* Imperil seedlings were used for transcriptome sequencing, with seedlings grown under normal conditions serving as controls (CK). Six RNA libraries (3 CK and 3 WT) were used for whole-transcriptome sequencing. The sequencing produced a total of 105.73 Gb of clean data, with a minimum of 93.75% of bases reaching Q30 in each sample (Additional file 2). After mapping to the *Secale cereale* reference genome (Secale cereale genome assembly Rye_Lo7_2018_v1p1p1 - NCBI - NLM (nih.gov), the proportions of mapped reads against the reference genome ranged between 91.72% and 92.58% for each sample (Additional file 2). The transcriptome sequencing revealed the expression of 22,020 genes. Novel gene discovery on the mapped reads resulted in the identification of 13,658 novel genes, including 4786 novel genes annotated with putative functions.

### Differential expression and enrichment analysis of DE-mRNAs responding to WS

**Fig. 2:**
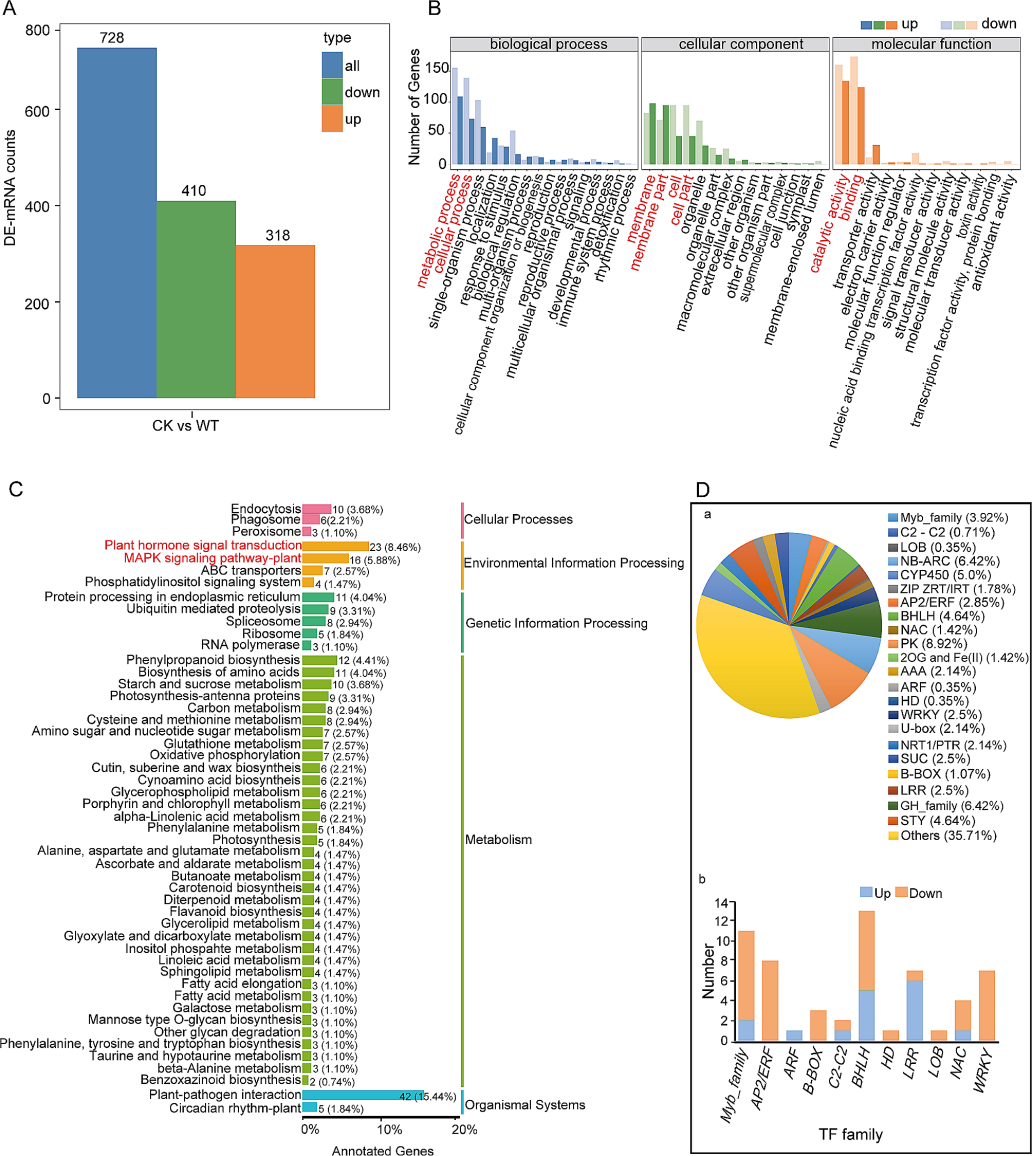
Gene expression and enrichment annotation of DE-mRNAs. Expressed DE-mRNAs **(A)**, GO classification annotation of DE-mRNAs (**B)**, KEGG pathway classification enrichment of DE-mRNAs (**C**), and annotated gene expression regulatory proteins and transcriptional factors of up-regulated DE-mRNAs (**D**)

The transcriptome analysis identified a total of 728 DE-mRNAs. Among these, 318 DE-mRNAs were upregulated, while 410 DE-mRNAs were downregulated (Fig. 2A, Additional file 3). To understand the functional relationships of the DE-mRNAs in terms of BP, MF, and CC. GO classification annotation was performed. Enriched BP terms were metabolic processes (GO:0008152), cellular processes (GO:0009987), and single-organism processes (GO:0044699). The enriched CC terms were membrane (GO:0016020), membrane part (GO:0044425), and cell (GO:0005623). Catalytic activity (GO:0003824), binding (GO:0005488), and transporter activity (GO:0005215) were the enriched MF (Fig. 2B). GO enrichment analysis of the upregulated DE-mRNAs was enriched in MF terms related to ADP binding, transmembrane transporter activity, zinc ion transmembrane transporter activity, and cysteine-type endopeptidase inhibitor activity involved in apoptotic process (Additional file 7A). Enriched CC terms were an integral component of membrane and RNA polymerase II transcription regulator complex (Additional file 7B). Enriched BP terms were defence response, glutamate catabolic process, lipid biosynthetic process, metal ion transport, and response to gibberellin (Additional file 7C). The KEGG pathway classification of the upregulated DE-mRNAs revealed significant enrichment in pathways related to plant hormone signal transduction, MAPK signaling pathway-plant, and plant-pathogen interaction (Fig. 2C). Also, KEGG pathway enrichment analysis of the upregulated DE-mRNAs was enriched in pathways related to plant-pathogen interaction, ABC transporter, diterpenoid biosynthesis, linoleic acid metabolism, and taurine and hypotuarine metabolism (Additional file 7D).

The GO terms that were enriched in comparison to the entire genome were further investigated. Enriched BP terms were protein-chromophore linkage (GO:0018298), photosynthesis, light harvesting in photosystem I (GO:0009768). The CC were enriched in photosystem I (GO:0009522) and photosystem II (GO:0009523). Enriched MF terms were chlorophyll binding (GO:0016168) and phenylalanine ammonia-lyase activity (GO:0045548). KEGG pathway classification of the upregulated and downregulated DE-mRNAs was performed to investigate their collective contributions to specific biological functions. The enriched annotated pathways were related to endocytosis (ko04144), plant hormone signal transduction (ko04075), protein processing in endoplasmic reticulum (ko04141), phenylpropanoid biosynthesis (ko00940), and plant-pathogen interaction (ko04626) (Fig. 2C). The DE-mRNAs were enriched in pathways related to taurine and hypotaurine metabolism (ko00430) (Additional file 9A). DE-mRNA activities associated with the enriched GO terms and KEGG pathways were analysed to investigate their specific roles in various biological functions. The analysis identified specific gene functions related to protein folding (GO:0006457), endomembrane system (GO:0012505), cell-cell junctions (GO:0005911), response to abiotic stimulus (GO:0009628), and the apoplast (GO:0048046) (Additional file 9B). Also, DE-mRNA KEGG pathways were involved in photosynthesis (ko00196) and photosynthesis antenna proteins (ko00196) (Additional file 9C).

Several transcription factors (TF) and expression regulatory proteins involved in plant stress responses were identified (Fig. 2D). These included the MYB superfamily, basic helix-loop-helix (bHLH) family, AP2/ERF family, leucine-rich repeat (LRR) proteins, C2-C2 domain, WRKY, no apical meristem (NAC) proteins, NB-ARC domain, protein kinase (PK) domain, cytochrome P450 (CYP450), ZIP zinc transporters (ZIP ZRT/IRT) family, serine/threonine/tyrosine (STY) protein kinase, POT family, glycosyl-hydrolase (GH) family, sugar transporters (SUC), and ATPase family. The up-regulated LRR and PK, which were involved in plant hormone signal transduction and plant-pathogen interaction. Auxin response factor (ARF), MYB, and bHLH were involved in plant hormone signal transduction. ZIP ZRT/IRT were involved in inorganic ion transport and metabolism. SUCs were involved in carbohydrate transport and metabolism. CYP450 and ABC transporters were involved in secondary metabolite biosynthesis, transport, catabolism, and defence mechanisms. The largest domains of the upregulated DE-mRNAs were the NB-ARC domains and the GH family. They were involved in plant-pathogen interaction, signal transduction, and carbohydrate transport metabolism. The highly clustered DE-mRNAs in the PPI were *SECCE7Rv1G0477800, SECCEUnv1G0539050, SECCE1Rv1G0001490*, and *SECCE2Rv1G0139380*. These genes were enriched in pathways related to protein processing in endoplasmic reticulum, signal transduction mechanisms, ubiquitin mediated proteolysis, RNA processing and modification, and RNA degradation. The enriched GO terms were ATP binding (GO:0005524), ATPase activity (GO:0016887), protein serine/threonine kinase activity (GO:0004674), ubiquitin-protein transferase activity (GO:0004842), poly(A)-specific ribonuclease activity (GO:0004535), cytoplasm (GO:0005737), CCR4-NOT complex (GO:0030014), chaperone cofactor-dependent protein refolding (GO:0051085), and protein refolding (GO:0042026) (Additional file 8).

### Identification and comparative analysis of lncRNAs and mRNAs responding to WS

**Fig. 3:**
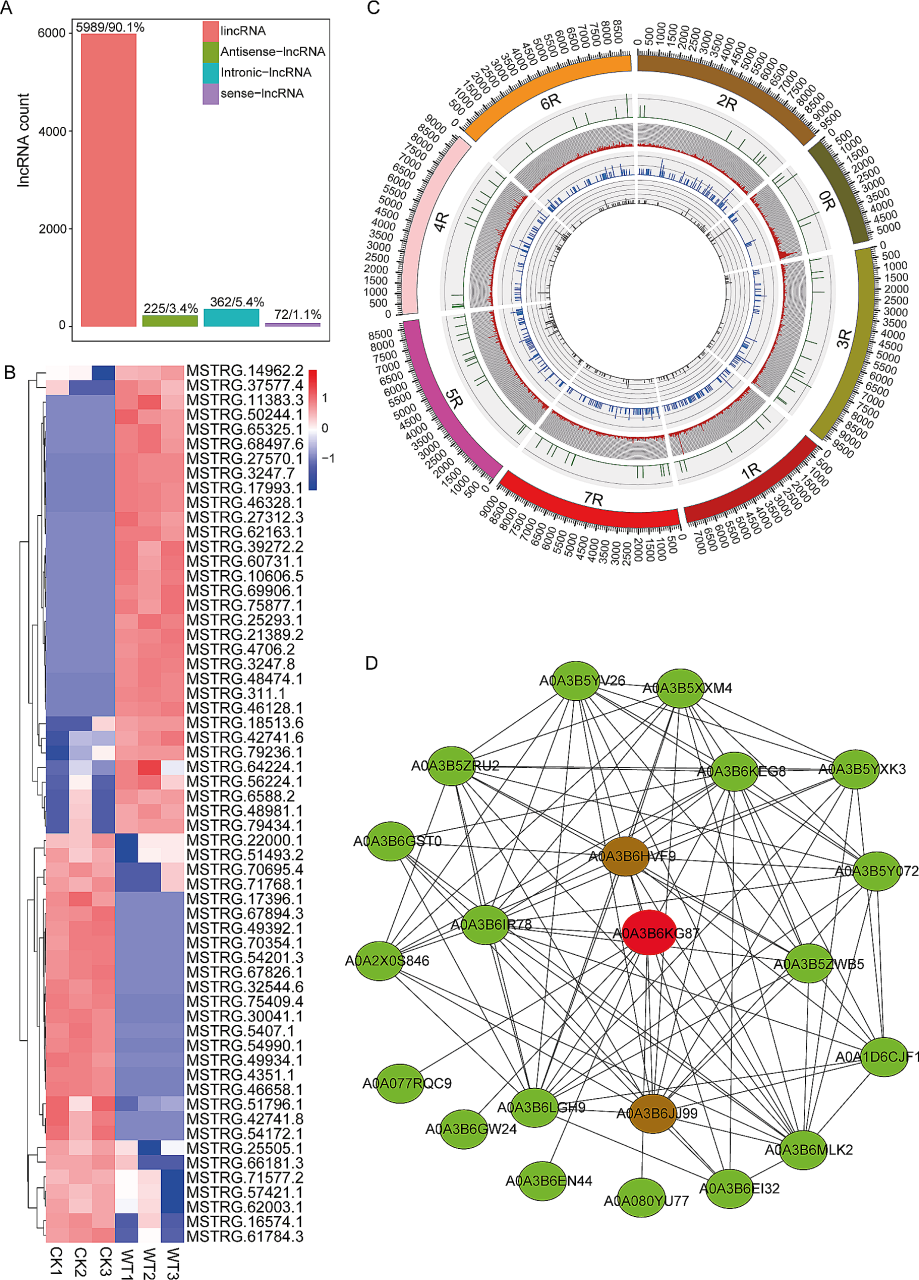
Expression, characterization, and network analysis of lncRNAs. Classification of lncRNAs **(A)**, hierarchical clustering of DE-lncRNAs **(B)**, classification of lncRNAs in the reference genome: sense-lncRNAs are shown by the green colour; red represents intergenic-lncRNAs; blue represents intronic-lncRNAs; and black represents antisense-lncRNAs **(C)**, and the protein-protein interaction network of DE-lncRNA target genes (**D)**

A total of 6648 lncRNAs were identified, including 32 upregulated DE-lncRNAs and 28 downregulated DE-lncRNAs (**Additional file 4**). The lncRNAs were classified based on their genomic locations as intergenic lncRNA (lincRNA), antisense lncRNA, intronic lncRNA, and sense lncRNA. The intergenic lncRNAs made up the largest category, accounting for 90.1% of the identified lncRNAs. 5.4% were intronic lncRNAs, 3.45% were antisense lncRNAs, and 1.1% were sense lncRNAs (Fig. 3A). The overall expression profiles of the DE-lncRNAs are shown in (Fig. 3B). The classified lncRNAs were visualised across the rye chromosomes to explore their distribution patterns. Intergenic lncRNAs were found across all chromosomes, with the highest density observed on the 0R, 4R, 1R, 5R, 6R, and 3R chromosomes. Intronic lncRNAs were highly distributed on the 2R, 1R, 7R, 5R, 4R, and 6R chromosomes. Sense lncRNAs showed a higher distribution on the 3R, 5R, 7R, and 4R chromosomes. Antisense lncRNAs were predominantly distributed on the 5R, 4R, and 6R chromosomes **(**Fig. 3C).

**Fig. 4:**
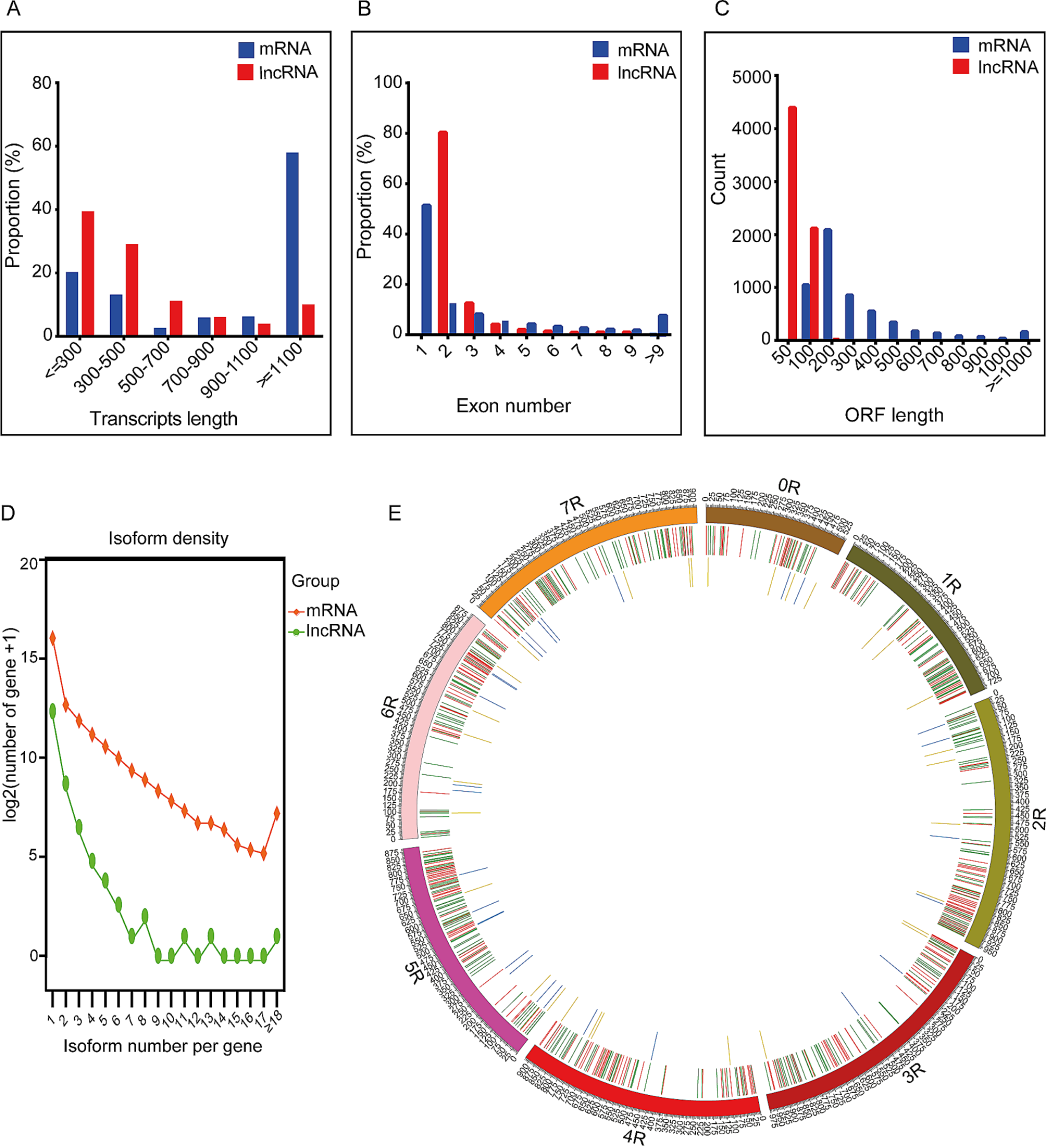
Comparative analysis of lncRNAs and mRNAs. Transcript length distribution **(A)**, number of exons **(B**), length of ORF **(C)**, alternative splicing event isoform diversity **(D)**, and visualisation of DE-mRNAs and lncRNAs in the reference genome (**E**): The outer circle represents the chromosomes based on the reference genome; the centre circle depicts the distribution of mRNAs on the chromosomes; and the inner circle represents the distribution of lncRNAs; upregulated DE-mRNAs are shown in red, while downregulated DE-mRNAs are shown in green. Upregulated DE-lncRNAs are highlighted in yellow, and downregulated DE-lncRNAs are highlighted in blue

Genomic comparative analysis between mRNAs and lncRNAs sequence lengths, open reading frame (ORF), and number of exons revealed that lncRNAs were shorter in length, with the majority ranging from 400 to 800 nt, compared to the mRNAs, which had the majority ranging from 400 to 2400 nt (Fig. 4A). Additionally, mRNAs had more exons compared to lncRNAs, and the distribution of mRNA exon numbers was higher than that of lncRNAs (Fig. 4B). Moreover, mRNAs possess a longer ORF, with a majority in the range of 100–500 nt compared to lncRNAs, which range from 50 to 100 nt (Fig. 4C). The isoform diversity of the mRNA and lncRNA transcripts originating from the samples in the alternative splicing event was investigated, and the isoform density was visualised to predict the abundance and relationships of different isoforms within the samples (Fig. 4D). Spatial arrangement of genes on chromosomes can have an impact on gene expression, and lncRNAs located near genes have the potential to act as promoters and regulate their expression [22]. The Circos visualisation tool [23] was used to visualise the relative positions of DE-lncRNAs and DE-mRNAs on the rye chromosomes. We observed a higher number of upregulated DE-mRNAs on the 5R, 4R, 3R, and 2R chromosomes. In contrast, the downregulated DE-mRNAs were predominantly distributed on the 7R, 2R, 5R, 3R, and 4R chromosomes. Additionally, there was a significant abundance of upregulated DE-lncRNAs on the 6R, 7R, 0R, 2R, and 4R chromosomes. The downregulated DE-lncRNAs were highly observed on the 5R, 6R, and 7R chromosomes (Fig. 4E).

### Annotation and enrichment analysis of DE-lncRNA target genes

**Fig. 5:**
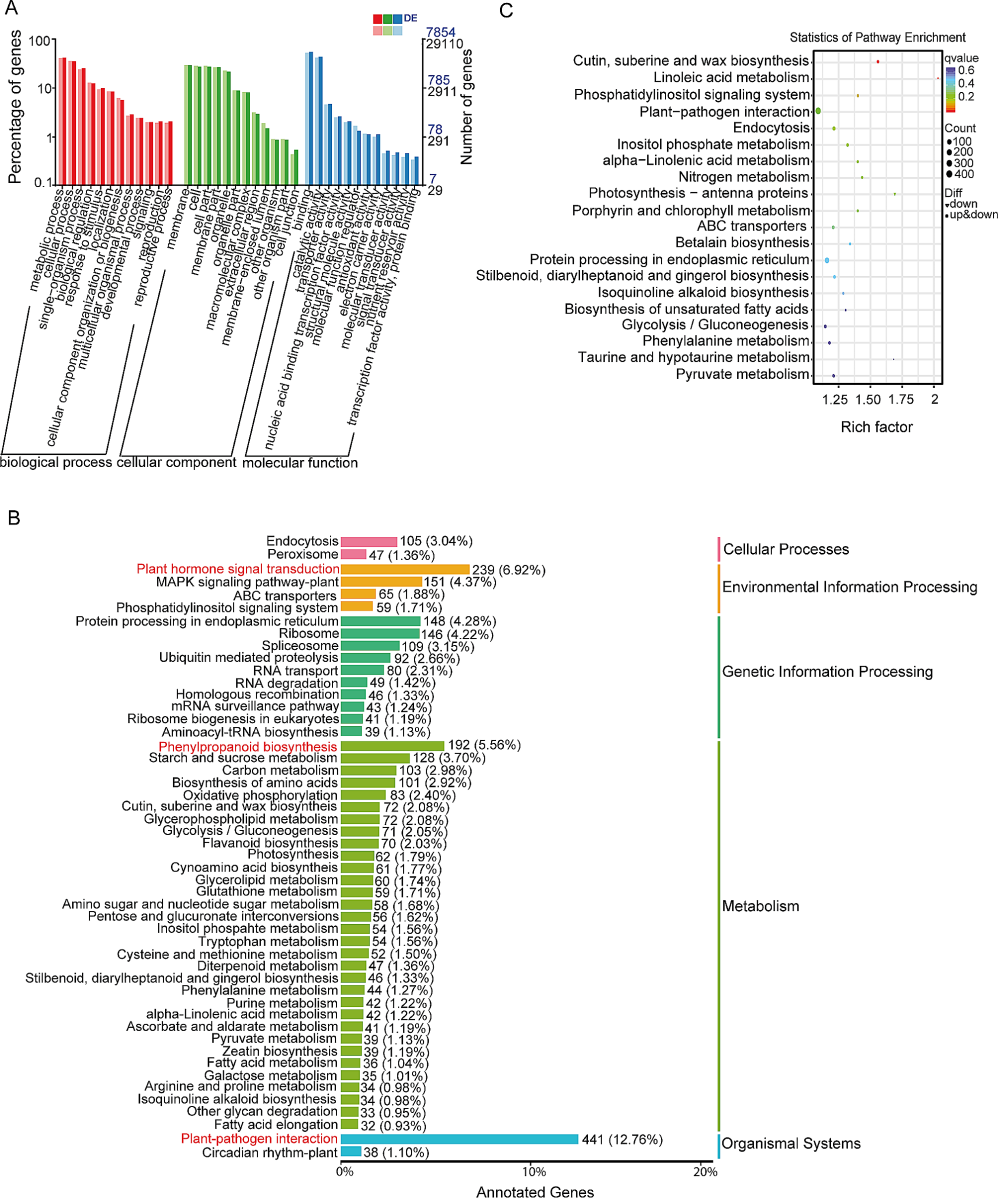
Classification and enrichment analysis of DE-lncRNA target genes. GO enrichment annotation of DE-lncRNA target genes (**A**), KEGG pathway classification annotation of DE-lncRNA target genes (**B**), and statistics of KEGG pathway enrichment of DE-lncRNA target genes (**C**)

To explore the regulatory functions of the DE-lncRNAs, GO annotation and KEGG pathway enrichment analyses on the DE-lncRNA target genes were performed. The GO annotation classification of the target genes revealed enriched BP terms related to metabolic processes (GO:0008152), cellular processes (GO:0009987), and single-organism processes (GO:0044699). The CC was enriched in the membrane (GO:0016020), cell (GO:0005623), and cell part (GO:0044464). Binding (GO:0005488), catalytic activity (GO:0003824), and transporter activity (GO:0005215) were enriched MF (Fig. 5A). GO functional enrichment study of the target genes discovered DNA integration (GO:0015074), (1-˃3)-beta-D-glucan biosynthesis process (GO:0006075), and oxylipin biosynthesis process (GO:0031408) as enriched BP. The CC was enriched in the 1,3-beta-D-glucan synthase complex (GO:0000148). Enriched MF terms were related to 1;3-beta-D-glucan synthase activity (GO:0003843), iron ion binding (GO:0005506), and oxidoreductase activity, acting on single donors with incorporation of molecular oxygen, incorporation of two atoms of oxygen (GO:0016702).

The KEGG pathway classification of the DE-lncRNA target genes was related to plant hormone signal transduction, phenylpropanoid biosynthesis, and plant-pathogen interaction as significantly enriched (Fig. 5B). The KEGG pathway enrichment analysis of the DE-lncRNA target genes revealed significant enrichment in pathways related to cutin, suberine and wax biosynthesis (ko00073) and linoleic acid metabolism (ko00591) (Fig. 5C). Clustering analysis of the GO terms and KEGG pathways of the DE-lncRNA *cis*- and *trans*-regulating genes influencing enrichment functions was performed. The activities of the *cis*-regulating genes were electron transporter, transferring electrons within the cyclic electron transport pathway of photosynthesis activity (GO:0045156) and cofactor binding (GO:0048037) (Additional file 10A), while the activities involved in the KEGG pathway functions were oxidative phosphorylation (ko00190) and photosynthesis (ko00195) (Additional file 10B). Moreover, the *trans*-regulating gene activities responsible for the GO term functions were the electron transporter, transferring electrons within cytochrome b6/f complex of photosystem II activity (GO:0045158) (Additional file 10C), and the KEGG pathway functional activities were fatty acid metabolism (ko01212), carotenoid biosynthesis (ko00906), peroxisome (ko04146), betalain biosynthesis (ko00965), and isoflavonoid biosynthesis (ko00943) (Additional file 10D).

To explore the coordinated function of the DE-lncRNAs in response to WS, a protein interaction analysis was performed on the target genes of the DE-lncRNA. The protein sequences of the DE-lncRNA target genes were blasted against *Triticum aestivum* protein sequences in the STRING database, and the ortholog proteins were used to build a PPI network for the DE-lncRNAs. A total of 21 proteins were involved in the main network based on the STRING database of *Triticum aestivum*. The guanylate kinase proteins *A0A3B6JJ99* and *A0A3B6HVF9*, and the glutamate-gated receptor protein *A0A3B6KG87*, were highly clustered in the network. These protein-enriched pathways were related to light-signal transduction, the metabolic pathway, and purine metabolism. GO enrichment analysis of these proteins revealed enriched terms related to ion transport (GO:0006811), integral component of membrane (GO:0016021), glutamate receptor (GO:0008066), the GMP metabolic process (GO:0046037), guanylate kinase activity (GO:0004385), ATP binding (GO:0005524), and phosphorylation (GO:0016310) (Fig. 3D).

### Co-expression association analysis of expressed mRNAs

**Fig. 6:**
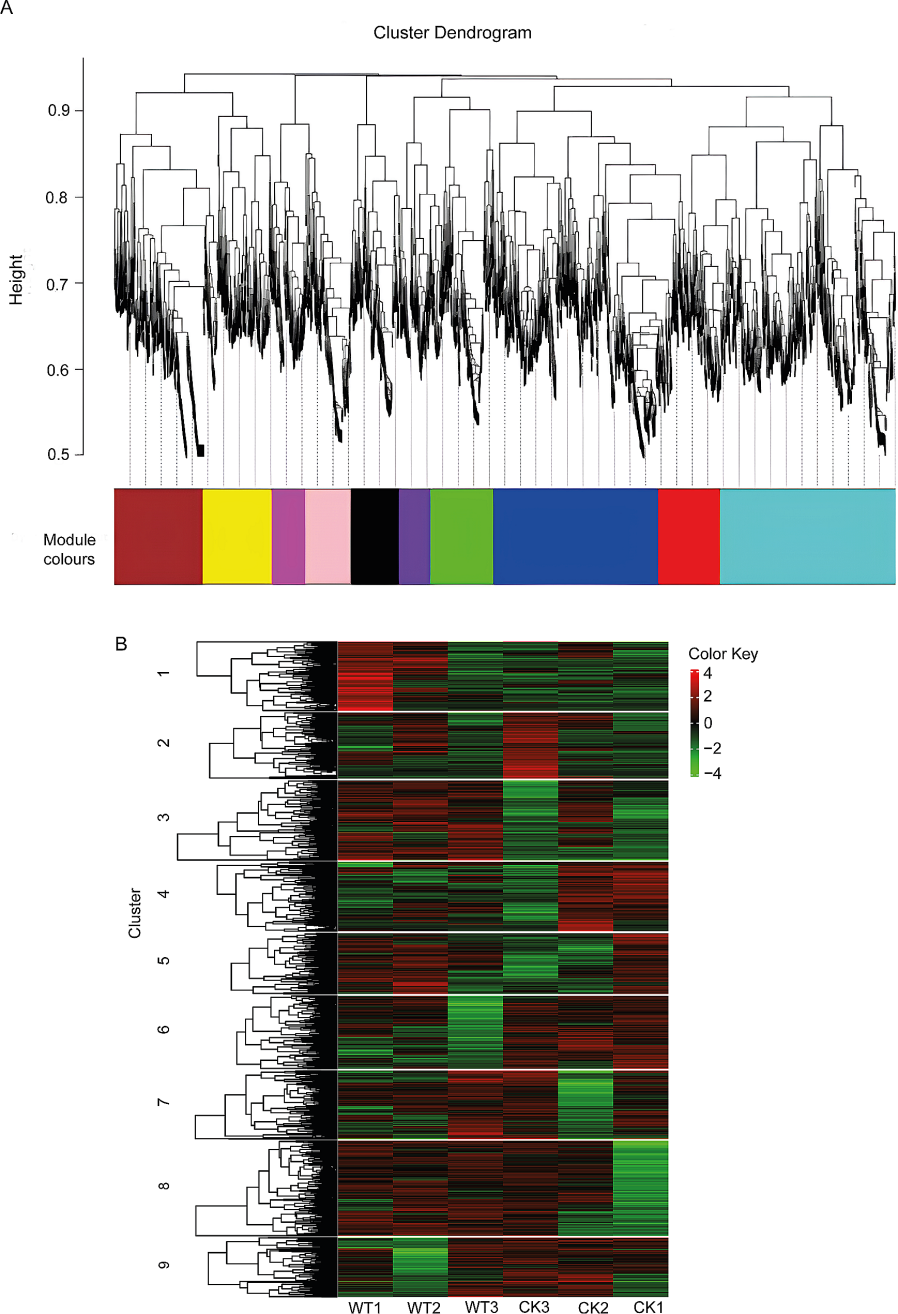
Co-expression association of mRNA-expressed genes. Expression module of mRNAs (**A**) and expression module clusters of mRNAs (**B**)

To investigate the relationships between co-expressed mRNAs and identify genes that were involved in specific regulatory functions, we utilised weighted correlation network analysis (WGCNA) using IDEP 1.12 [24] on highly reliable transcripts. We grouped genes with similar expression into modules to explore how they are related in response to WS. A soft threshold of 5 and a minimum module size of 100 were used, resulting in 10 distinct modules. Each module was significantly associated with WS response. The pathways that were enriched in the modules include anthocyanin biosynthesis, glutathione metabolism, plant hormone signal transduction pathways, MAPK signaling pathway-plant, secondary metabolite biosynthesis, phenylpropanoid biosynthesis, oxidative phosphorylation, plant-pathogen interaction, protein processing in endoplasmic reticulum, and ubiquitin-mediated proteolysis (Fig. 6A). A total of 3000 mRNAs were associated with these 10 modules, with the number ranging from 120 in the ‘purple’ module to 671 in the ‘turquoise’ module (Additional file 5). The modules were grouped into nine clusters, with the clusters containing 3000 genes and ranging from 278 to 445 genes in each cluster (Fig. 6B). To investigate the potential functions of the genes within the modules, we performed an enrichment analysis to identify the genes that encode a specific group of proteins within each module. A total of 43 genes encoding proteins that are known to be associated with plant stress regulation were identified. Among these modules, the blue and turquoise modules contained the highest number of genes (Additional file 11).

GO enrichment analysis was conducted on the clusters to identify the significantly enriched GO terms and the genes enriched in each term. There was no significant enriched term in cluster 6. Genes in cluster 1 were enriched in heme binding, tetrapyrrole binding, iron ion binding, oxidoreductase activity, monooxygenase activity, oxidoreductase activity acting on paired donors with incorporation or reduction of molecular oxygen, transition metal ion binding, nicotianamine metabolic process, nicotianamine biosynthetic process, and tricarboxylic acid biosynthetic process. Genes in cluster 2 were enriched in regulation of cellular respiration, regulation of the generation of precursor metabolites and energy, and ADP binding. In cluster 3, the enriched terms were oligopeptide transport, peptide transport, amide transport, dipeptide transport, tripeptide transmembrane transporter activity, dipeptide transmembrane transporter activity, iron ion binding, oligopeptide transmembrane transporter activity, and peptide transmembrane transporter activity. Cluster 4 was enriched in lyase activity, carboxy-lyase activity, carbon-carbon lyase activity, pyridoxal phosphate binding, and vitamin B6 binding. In cluster 5, the enriched terms include protein phosphorylation, protein kinase activity, phosphorylation, adenyl nucleotide binding, adenyl ribonucleotide binding, phosphotransferase activity alcohol group as acceptor, nucleotide binding, kinase activity, small molecule binding, and anion binding. Genes in cluster 7 were enriched in protein phosphorylation, phosphorus metabolic process, phosphate-containing compound metabolic process, protein kinase activity, transferase activity transferring phosphorus-containing groups, adenyl nucleotide binding, adenyl ribonucleotide binding, phosphotransferase activity alcohol group as acceptor, and kinase activity. Cluster 8 was enriched in hydrolase activity hydrolyzing O-glycosyl compounds, cysteine-type peptidase activity, hydrolase activity acting on glycosyl bonds, beta-fructofuranosidase activity, UDP-glycosyltransferase activity, extracellular region, glycosyltransferase activity, and the apoplast. ADP binding was enriched in cluster 9 (Additional file 6).

### Validation, conserved motif, and *cis*-regulatory element analysis of DEGs

**Fig. 7:**
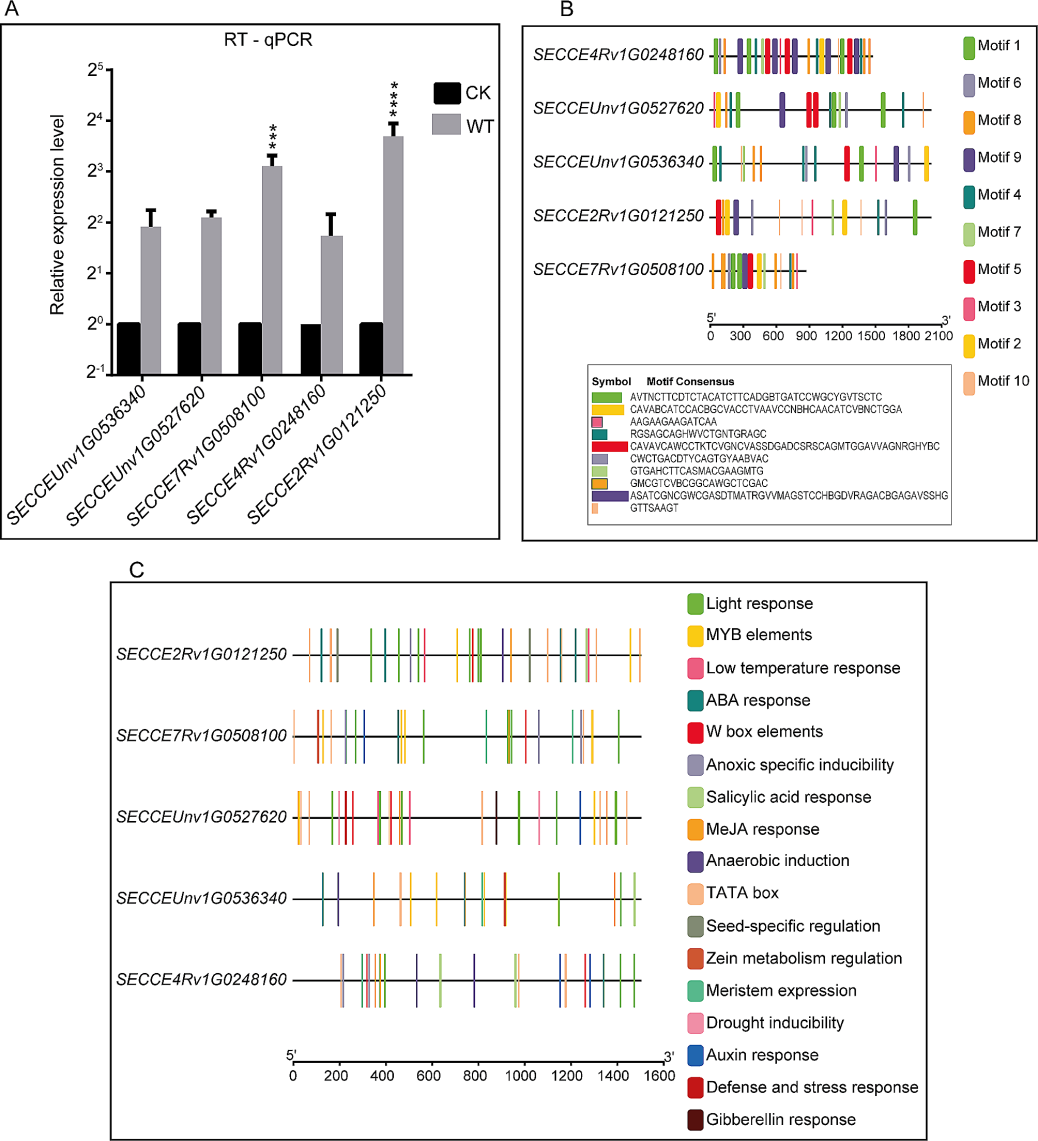
Sequencing validation and motif discovery. RT–qPCR analysis of selected upregulated DE-mRNAs (**A**), conserved motifs in the exons of the selected DE-mRNAs **(B)**, and *cis*-acting regulatory elements in the promoter region of the selected DE-mRNAs **(C)**. Asterisks represent significant differences between three biological replicates at *p* = 0.0005 (***) and *p* < 0.0001 (****)

To confirm the sequencing results, the expression levels of 5 selected DE-mRNAs were analysed using qRT-PCR. Among the selected DEGs, *SECCE2Rv1G0121250* had a higher level of expression, followed by *SECCE7Rv1G0508100*. *SECCEUnv1G0527620*, *SECCEUnv1G0536340*, and *SECCE4Rv1G0248160* exhibited progressively lower expression levels (Fig. 7A). *SECCE2Rv1G0121250* was annotated as an NB-ARC domain, *SECCE7Rv1G0508100* as a glycosyl hydrolase family 16, *SECCEUnv1G0527620* as an ABC-2 type transporter, *SECCEUnv1G0536340*, and *SECCE4Rv1G0248160* as a protein kinase domain. Motifs play an important role in the interaction of various modules within transcriptional complexes and signal transduction [25]. The structure of a motif is connected with gene classification. The MEME suite was used to analyse 10 conserved motifs present in the exons of the selected DE-mRNAs. Each motif occurs once per gene. (Fig. 7B). The *cis*-acting regulatory elements in the promoter region of the selected genes revealed several stress-responsive elements involved in WS response, such as anoxic-specific inducibility, anaerobic induction, gibberellin response, low temperature response, MYB elements, MeJA response, ABA response, auxin response, and salicylic acid response, as well as numerous light-responsive elements and elements associated with plant growth and development, including meristem expression, zein metabolism regulation, cell cycle control, seed-specific regulation, TATA-box, and W box elements. (Fig. 7C).

## Discussion

Waterlogging negatively affects crop growth and productivity. To gain a better understanding of mRNA and lncRNA responses to WS in rye, whole-genome transcriptome sequencing of WS-treated rye was conducted to investigate the key regulatory genes and pathways involved in rye WS responses. The results of this study indicate a response to WS by using diverse metabolic, ROS scavenging, and energy regulation processes.

### Carbon metabolism and oxidative phosphorylation regulations under WS

When plants experience hypoxic conditions, they promptly adjust their gene transcription to produce anaerobic polypeptides through processes such as glycolysis and fermentation [26]. A study conducted by Teaoh et al. [27] found that genes associated with carbon metabolism, starch metabolism, and glycolysis showed significant upregulation after one day of waterlogging. However, our present study is inconsistent with the previous research. We found that genes related to carbon metabolism and starch and sucrose metabolism were upregulated, while 2 genes associated with glycolysis, *SECCE5Rv1G0332040* and *SECCE6Rv1G0387240*, were downregulated. This indicates that mRNAs may activate metabolic adaptations that enable rye to adapt to anaerobic conditions and sustain important metabolic functions. Under low oxygen conditions, anaerobic respiration occurs in the roots and channels glycolysis towards fermentative pathways. Studies by Teaoh et al. [27] and Ren et al. [28] discovered that genes related to the tricarboxylic acid (TCA) cycle were downregulated during WS. However, our study produced conflicting results, as we found an upregulated gene, *SECCE2Rv1G0108460*, associated with the TCA cycle. Additionally, we found the upregulation of genes related to oxidative phosphorylation. These findings suggest that rye may activate mechanisms to overcome energy limitations and maintain essential cellular functions during WS.

### Adjustments of chlorophyll content and photosynthetic activity in response to WS

A study has demonstrated that WS can hinder the transport of sucrose in plants, leading to an accumulation of sucrose and soluble sugars in the leaves. This study found that the downregulation of two genes associated with sucrose breakdown, such as sucrose synthase 4 and beta glucosidase 11, may contribute to the increase in sucrose content [29]. However, our current data contradicts this previous study. We identified 2 upregulated beta glucosidase genes (*SECCE5Rv1G0338290* and *SECCE3Rv1G0210200*) and 1 downregulated beta glucosidase gene (*SECCE5Rv1G0353020*). This suggests that sucrose transportation may not be completely inhibited, and sucrose breakdown may occur in rye to support the tissues that cannot undergo photosynthetic activity. Furthermore, it indicates a potential reconfiguration of the photosynthetic machinery to optimize energy efficiency and sustain the vitality of rye under WS [30].

### Phenylpropanoid biosynthetic pathway metabolites change under WS

When plants encounter stress conditions, the phenylpropanoid biosynthetic pathway is activated as a means of response to the stress conditions. It has been observed that the expression of genes involved in phenylpropanoid biosynthesis is altered, and the accumulation level of phenylpropanoids is modified in response to cold, drought, flood, and salt stress in *Ocimum tenuiflorum* II [31]. Furthermore, a study has demonstrated that WS leads to increased expression of genes associated with phenylpropanoid biosynthesis in *Oryza sativa* [32]. Consistent with these studies, our current study also discovered a similar pattern, where upregulated DE-lncRNA target genes and DE-mRNAs were significantly enriched in the phenylpropanoid biosynthesis pathway (Figs. 2C and 5B). This indicates that the phenylpropanoid biosynthetic pathway plays a significant role in the rye response to WS [6].

### Carotenoid biosynthesis adjustments under WS

Carotenoids play essential roles in various plant processes and act as potential antioxidants during plant stress. They have multiple functions, such as capturing light energy, neutralising harmful byproducts generated by chlorophyll, and protecting plants from oxidative damage. The levels of carotenoids can be influenced by WS, although the specific response varies depending on the plant species, as well as the duration and intensity of the stress period [33]. Studies have reported both an increase and a decrease in carotenoid levels among 24 different genotypes of sugarcane [34] and four cultivars of mung bean [35]. In our current study, we observed that upregulated DE-lncRNA target genes and 2 DE-mRNAs were enriched in carotenoid biosynthesis, which is responsible for synthesising carotenoids during plant stress (Additional file 10D, Additional file 9C ). Based on this result, we conclude that WS increases carotenoid levels in rye and is potentially associated with the activation of the xanthophyll cycle [6, 36].

### ABC transporter and MAPK signaling pathway regulations under WS

The MAPK signaling pathway mediates the production, signaling, and transport of phytohormones [37]. During WS, various proteins involved in hormone metabolism, signal transduction, transcriptional regulation, glucose degradation/sucrose accumulation, suppression of ROS scavenging, and ubiquitin-mediated proteolysis are expressed at different stages. Plants maintain a balance between phytohormone synthesis and transportation while controlling their responses to waterlogging through complex signaling pathways [38]. In the current study, upregulated DE-mRNAs were enriched in pathways related to plant hormone signal transduction, the MAPK signaling pathway-plant, and plant-pathogen interaction (Fig. 2C). Among these DE-mRNAs, 12 were annotated to be involved in the plant hormone signal transduction pathway, including a highly expressed ARF TF *SECCE5Rv1G0301670*, located on the 7R chromosome. Interestingly, this ARF TF was the only differentially expressed ARF TF identified in this study. This suggests that the ARF TF may play a significant role in enhancing ethylene production and activating signal transduction mechanisms during the early response of rye to WS [39]. The upregulated DE-mRNAs in this study showed significant enrichment in the MAPK signaling pathway-plant and ABC transporter pathways. This indicates that the activation of the MAPK signaling pathway-plant could potentially trigger the signaling of important hormones. These hormones were then likely transported by ABC transporters, facilitating their distribution to waterlogged regions and activating defence responses [37, 39].

### Regulation of waterlogging-responsive genes by TFs

In a study by Yu et al. [6], several upregulated genes involved in the ethylene-response related pathway were identified, including a gene encoding a RING/U-box superfamily protein. In our study, we observed the differential expression of 3 genes encoding the U-box domain, both upregulated and downregulated **(**Fig. 2D). This finding suggests that the U-box domain may also contribute to the ethylene signaling pathway by regulating early responses to WS. Studies conducted by Li et al. [40] and Yu et al. [6] on maize and wheat identified several upregulated TFs involved in flooding stress, including bHLH, NAC, MYB-related, WRKY, C2H2, and AP2/ERF. Interestingly, these results are not consistent with the findings of our present study. In our current study, all the differentially expressed WRKY and AP2/ERF TFs were downregulated (Fig. 2D). This suggests that the genes regulated by these TFs are suppressed under WS in rye, suggesting that the activation of ethylene production genes as an early response to WS in rye may be mediated by ARF, the U-box domain, and other signaling pathways, rather than directly by AP2/ERF and WRKY TFs [39].

### Lipid biosynthetic process adjustments under WS

In a study conducted by Xie et al. [41], they found that WS induced significant changes in mRNA abundance in the genes involved in lipid biosynthesis and metabolism. They discovered that 48-h light submergence treatment repressed the transcripts of genes in fatty acid synthesis, but significantly induced genes related to the fatty acid degradation pathway. Our results contradict this study, our data showed that *SECCE1Rv1G0011490*, *SECCEUnv1G0538000*, and *SECCEUnv1G0538000* were upregulated genes related to fatty acid synthesis. Also, the upregulated fatty acid degradation gene *SECCE1Rv1G0011490* was annotated. This suggests that under WS, rye employs complex and context-dependent regulation of lipid metabolism. Additionally, Xie et al. [41] study observed significant changes in the mRNA levels associated with sphingolipid metabolism during WS. Specifically, proteins involved in the synthesis and breakdown of ceramides and sphingolipid long-chain bases were upregulated in response to WS. Our current data is in agreement with this study, we found upregulated DE-mRNAs related to ceramides and sphingolipid metabolism. This finding suggests that sphingolipids are likely involved in rye response to WS [41].

### Regulations of glutamate and gibberellin under WS

Glutamate undergoes a decarboxylation reaction to be converted into gamma-aminobutyric acid (GABA) [42]. This conversion is important for regulating plant responses to both biotic and abiotic stresses, as well as maintaining the balance between carbon and nitrogen metabolism [43]. Gibberellins play a significant role in regulating shoot elongation and the formation of adventitious roots in plants. They work in synergy with auxin and oppose the effects of abscisic acid (ABA) in controlling the growth of adventitious roots and shoot/internode elongation [44]. In our present study, we found that upregulated DE-mRNAs were enriched in response to gibberellin and the glutamate catabolic process (Additional file 7C). This suggests that glutamate, which is known to increase chlorophyll content and the rate of photosynthetic activity, plays an important role in rye WS response [45].

### Cytochrome b6/f complex metabolic process under WS

Cyclic electron transfer/flow (CET/CEF) is important for efficient photosynthesis as it maintains the appropriate ATP/NADPH ratio required for various regulatory and metabolic processes [46]. The NADH dehydrogenase-dependent CEF pathway relies on photosystem I and cytochrome b6/f [46]. Cytochrome b6/f plays a central role in the light-dependent processes of oxygenic photosynthesis, acting as a bridge between photosystem II and photosystem I. It facilitates the oxidation and reduction of electron carriers, namely plastoquinol and plastocyanin, which are essential components involved in electron transport, redox balance, ROS scavenging, stress signaling, gene expression, and energy allocation within the photosynthetic machinery of plants [47]. In our current study, we observed that both *cis*- and *trans*-regulated DE-lncRNAs were significantly enriched in activities related to the transporter, transferring electrons within the cyclic electron transport pathway of photosynthesis, as well as the electron transporter, transferring electrons within the cytochrome b6/f complex of photosystem II (Additional file 10A, Additional file 10C). These findings suggest that lncRNAs may activate mechanisms that facilitate efficient photosynthetic processes in rye under WS.

### Regulations of diverse metabolic pathways under WS

Peroxisomes play an important role in various metabolic processes within plants. These organelles are responsible for important functions such as the breakdown of fatty acids through β-oxidation, the glyoxylate cycle in seedlings, and photorespiration in leaves [48]. They house antioxidant enzymes and enzymes involved in generating ROS, or reactive nitrogen species, which are essential for maintaining redox homeostasis. The ROS signals originating from chloroplasts, mitochondria, and peroxisomes are believed to converge in the cytoplasm and connect to the MAPK signaling pathway, ultimately regulating the expression of nuclear genes [49]. In our study, we found that DE-lncRNA target genes were significantly enriched in peroxisome-related processes, fatty acid metabolism, and the MAPK signaling pathway-plant (Additional file 10D). These findings suggest that under WS, lncRNAs induce interconnected responses that involves lipid breakdown, ROS detoxification, and hormone signaling in rye. A study by Li et al. [40] revealed that various pathways, including flavonoid biosynthesis, biosynthesis of secondary metabolites, taurine and hypotaurine metabolism, cutin, suberine and wax biosynthesis, and glutathione metabolism, were significantly enriched by DEGs under different flooding conditions. In line with these findings, our study also showed enrichment of upregulated DE-mRNAs in these pathways (Fig. 2C, Additional file 9C, Additional file 7D). This suggests that the activation of these pathways may be an important mechanism involved in rye response to WS [32]. A study conducted by Yu et al. [6] identified downregulated genes associated with benzoxazinoid biosynthesis and alpha-linolenic acid metabolism pathways. Consistently, our current study also discovered significant enriched DE-mRNAs and DE-lncRNA target genes involved in these pathways, both upregulated and downregulated. This suggests that these pathways may play an important role in regulating rye response to WS. In addition, upregulated DE-mRNAs were predominantly NB-ARC domains. These domains are known to play an important role in the plant-pathogen interaction pathway, indicating the involvement of this pathway in diverse intracellular immune receptors [50].

### Oxidative stress and antioxidant changes in the leaves of waterlogged rye

Plant growth and development can be enhanced by γ-aminobutyric acid (GABA) under WS. GABA increases the photosynthetic rate, chlorophyll content, and antioxidant enzyme activities such as SOD, POD, and CAT, which play a key function in reducing ROS levels and lipid peroxidation while decreasing MDA content to protect plant cells from membrane damage and oxidative stress caused by WS [45]. In this study, stress symptoms were observed in all the stressed seedlings, although the severity varied among different species. Among them, *S. cereale* L. Imperil exhibited the mildest symptoms. A study by Li et al. [51] observed that the activities of SOD, POD, and CAT increase under WS to efficiently regulate superoxide anion and H_2_O_2_. Consistent with the present study, the activity of CAT, POD, and SOD increased significantly in all the WS samples (Fig. 1C-E), suggesting that antioxidant activities were increased in all the species. Teaoh et al. [27] also observed that MDA levels decreased significantly under WS. In the present study, we observed that the MDA level in each cultivar increased with the exception of *S. cereale* L. Imperil which had a significant decrease under WS (Fig. 1B), suggesting that *S. cereale* L. Imperil exhibited lower levels of oxidative damage. These findings suggest that *S. cereale* L. Imperil exhibits better tolerance to WS and may have reduced susceptibility to lipid peroxidation. Furthermore, it demonstrates a strong capacity to mitigate oxidative stress by efficiently breaking down hydrogen peroxide and scavenging ROS [52].

### WGCNA network and genomic comparison of mRNA and lncRNA under WS response

Co-expression analysis is used to cluster genes based on their shared expression patterns across multiple samples. The resulting gene modules often reflect specific biological processes. Our study found that all 10 modules were significantly enriched in pathways related to plant regulation of WS, including anthocyanin biosynthesis, glutathione metabolism, MAPK signaling pathway-plant, oxidative phosphorylation, phenylpropanoid biosynthesis, plant-pathogen interaction, ubiquitin-mediated proteolysis, and secondary metabolite biosynthesis (Fig. 6A). These findings indicate that rye utilises complex and coordinated response mechanisms involving pigment production, antioxidant activity, stress signaling, energy regulation, and secondary metabolite synthesis to effectively regulate WS in rye.

The genomic comparisons between mRNAs and lncRNAs in our study were consistent with previous findings on sea cucumbers [53] and cultivated peanuts [54]. These comparisons found that lncRNAs exhibit shorter transcript lengths, fewer exons, shorter open reading frames (ORFs), and lower expression levels compared to mRNAs (Fig. 4A-C). Furthermore, we observed that the promoter region of selected DEGs contains several stress-responsive elements (Fig. 7C). This suggests the association of DE-mRNAs with diverse regulatory functions in rye growth and development under WS, highlighting their role in the plant’s response to water stress.

## Conclusions

In this study, 4 rye cultivars and 2 wild species were waterlogged, and one cultivar (*Secale cereale* L. Imperil) showed relative tolerance to WS. Subsequently, transcriptome sequencing was performed on this cultivar, and lncRNAs and mRNAs involved in WS responses were investigated. We discovered that rye response to WS involves several biological processes, such as carbohydrate and photosynthetic activity adjustments, energy regulation, and scavenging ROS. Sucrose transportation was not inhibited, and photosynthetic machinery was active under WS. Carotenoid levels and the activities of the tricarboxylic acid cycle increase under WS. Rye effectively regulates antioxidant enzyme activities and mitigates oxidative stress under WS. Sucrose transportation was not significantly inhibited, and sucrose breakdown occurred in rye under WS. These findings suggest that mRNA and lncRNA utilise a complex range of biological processes to regulate WS in rye. This study offers a comprehensive resource for studying the lncRNA and mRNA regulatory mechanisms in rye, which are important for breeding purposes.

## Methods

### Plant materials and growth conditions

The study utilised four rye cultivars (*Secale cereale* L. Imperil, *S. cereale* L. Austria, *S. cereale* L. KingII, and *S. cereale* L. Shengli), and two wild species (*S. strictum* (ADAMS) and *S. vavilovii* (PI618682, Poland)). The seeds of these cultivars were planted in plastic pots and grown in a temperature-controlled growth room with an average humidity of 60%. The growth conditions included a 28^o^C/22^o^C day/light cycle and a 14-hour light/10-hour dark cycle, with the light period lasting from 7:00 am to 9:00 pm. The treatment was performed at 8:00 am, following one hour of illumination. After 18 days, the seedlings were submerged in water, and the water level was maintained approximately 2–3 cm above the soil throughout the treatment. Control seedlings, which did not receive any treatment, were also grown under the same conditions. After 12 days of WS treatment, the leaves of the seedlings from each plot were collected. For both the treatment and control groups, three replicates were sampled for total RNA isolation. These samples were snap-frozen in liquid nitrogen and stored at -80 °C.

### Physiological index analysis

The malondialdehyde (MDA) content and antioxidant enzyme activity assays, such as superoxide dismutase (SOD), peroxidase (POD), and catalase (CAT), in the leaves of each of the samples were measured following the established methods [55, 56].

### Statistical analysis

Statistical analysis was performed using Genstat 22.1 (VSN International Ltd.). A one-way ANOVA was conducted to assess the differences between the control and waterlogged samples. Significant differences at the 0.05 probability level were identified using the Duncan’s Multiple Range Test (DMRT).

### RNA extraction, library preparation, and assessment for sequencing

Following the manufacturer’s instructions, total RNA was extracted from the *S. cereale* L. Imperil leaves using TRIzol reagent (Invitrogen, Gaithersburg, MD, USA). RNA integrity was examined using 1.5% agarose gel, and the concentration and purity of the RNA were determined using a NanoDrop 2000 spectrophotometer (Thermo Fisher Scientific, USA). The NENext^R^Ultra^TM^Directional RNA Library Prep Kit for Illumina^R^(NEB, USA) was then used to create libraries from high-quality RNA samples using the manufacturer’s instructions. At Biomarker Technologies Corporation, Beijing, China, transcriptome sequencing of the prepared libraries was carried out using an Illumina NovaSe q6000 system, and paired-end 150-bp reads were generated.

### Quantification of gene expression level of mRNAs and lncRNAs in the transcriptome

The study generated a total of six RNA-Seq datasets. To ensure data quality, the raw reads deposited into the SRA database (accession number: PRJNA1014184) were evaluated using the FastQC programme (V0.11.3) [57] and filtered using Trimmomatic (v0.38) [58] to obtain clean data. The clean reads were aligned to the *S. cereale* L. genome (Assembly: GCA_000751015.1) using HISAT2 (v2.0.5) [59] with the default parameters. The gene expression level was calculated as Fragments Per Kilobase of transcript per Million fragments mapped (FPKM) using StringTie (1.3.1) [60] and normalised by log10. Coding Potential Calculator [61], Coding Non-Coding Index [62], Coding Potential Assessment Tool [63], and Pfam [64] were used to identify and classify the lncRNAs, and lncRNAs found in only one sample were eliminated. New transcripts were predicted using Cufflinks (v2.2.0) [65], with selection criteria including a length greater than 200 bp, at least two exons, and a coverage degree of three or higher.

### Differential gene expression and structural analysis

Differential expression analysis was carried out using the DESeq2 R package [66]. *P*-values were assigned to each gene, and these p-values were then converted into q-values using the Benjamini-Hochberg method to control for the false discovery rate [67]. Significantly differentially expressed genes were identified using thresholds of |log2FoldChange| ≥1 and q-value < 0.05. ASprofile [68] was used to obtain the AS categories and corresponding expression levels for each sample.

### Gene classification, enrichment, and protein-protein interaction analysis

The GO (http://www.geneontology.org/) [69] and KEGG pathway (http://www.genome.jp/kegg/) [70] databases were used for classification analysis of the DE-lncRNA target genes and DE-mRNAs [70]. KOBAS software was used for statistical significance analysis of the DE-mRNAs and DE-lncRNA-target genes [71]. The ClusterProfiler tool was utilised to perform GO enrichment and clustering analysis of the DE-mRNAs and DE-lncRNA-target genes [72]. The PPI of the DE-lncRNA target genes and DE-mRNAs was constructed using a BLAST search (BLASTX) to align their protein sequences to *Triticum aestivum* in the STRING database (http://string-db.org/) [73]. The protein-interaction relationship of the orthologs was used to create an interaction network for the DE-lncRNA target genes and DE-mRNAs. The predicted PPIs were then visualised using Cytoscape [74].

### Quantitative real-time PCR (RT-qPCR)

The FastPure Universal Plant Total RNA Isolation Kit was used for RNA extraction from the leaves following the manufacturer’s instructions, and single-strand cDNA was synthesised using the HiScript^R^ II 1^st^ Strand cDNA Synthesis Kit (Nanjing Vazyme Biotech Co., Ltd., China) according to the manufacturer’s protocol. ChamQ SYBR qPCR Master Mix (Vazyme Biotech Co., Ltd., Nanjing, China) was used to perform qRT-PCR using gene-specific primers, and the expression data were normalised with an internal reference gene (Additional file 1) in a 20 µl reaction. The thermal profile used is as follows: stage 1: 95 °C for 2 min, stage 2: 95 °C for 10 s, 60 °C for 30 s for 40 cycles; and stage 3: 95 °C for 15 s, 60^o^C for 1 min, 95^o^C for 15 s. Melting curve analysis was used to show how specific the amplification products were, and the 2^− ΔΔCT^ method was used to calculate the relative expression levels of the genes [75]. Three biological replicates were processed for each. GraphPad Prism software was used to analyse the data [76], with a p-value ≤ 0.05 considered statistically significant.

### Discovery of conserved motif and ***cis***-regulatory elements

The promoter sequence of randomly selected DE-mRNAs was retrieved from the EnsemblPlants database (https://plants.ensembl.org/index.html). A total of 1500 bp upstream regions were submitted to the PlantCARE database (accessed on January 1 2024) to identify the *cis*-acting regulatory elements [77]. The MEME suite (http://meme-suite.org/index.html) was used to analyse the conserved motifs of the exon region of the selected DE-mRNAs [78]. Only motifs with an e-value < 1e − 20 were retained and visualised using TBtools software [79].

### Electronic supplementary material

Below is the link to the electronic supplementary material.

**Additional file 1**. Gene-specific primers used for RT-qPCR analysis in this study.

**Additional file 2**. Summary of RNA sequencing results.

**Additional file 3**. Expression results of differentially expressed mRNAs.

**Additional file 4.** Expression results of differentially expressed lncRNAs.

**Additional file 5.** Co-expression association cluster module of expressed mRNAs.

**Additional file 6.** Enrichment pathways of co-expression clustered genes.

**Additional file 7.** Enrichment analysis of upregulated DE-mRNAs. Enriched MF (A), enriched CC (B), enriched BP (C), and enriched KEGG pathways (D).

**Additional file 8**. Protein-protein interaction network of DE-mRNAs

**Additional file 9.** Enrichment and clustering analysis of DE-mRNAs. Statistics of KEGG pathway enrichment of DE-mRNAs (A), GO term enrichment clustering of DE-mRNA targeted genes (B), and KEGG pathway enrichment clustering of DE-mRNA targeted genes (C). The terms and pathways with higher gene expression levels are shown in red, while those with lower levels are shown in blue; the number of enriched genes associated with each term and pathway is indicated in brackets.

**Additional file 10**. Enrichment analysis of DE-lncRNA target genes. GO-term enrichment clustering of DE-lncRNA *cis*-target genes (A), KEGG pathway enrichment clustering of DE-lncRNA *cis*-target genes (B), GO-term enrichment clustering of DE-lncRNA *trans*-target genes (C), and KEGG pathway enrichment clustering of DE-lncRNA *trans*-target genes (D).

**Additional file 11.** Description of genes encoding specific proteins in the modules.

## Data Availability

The sequencing data that support the findings of this study have been deposited in the National Center for Biotechnology Information (NCBI) Sequence Read Archive and are accessible through SRA accession number PRJNA1014184 at https://www.ncbi.nlm.nih.gov/sra/?term=PRJNA1014184.
