# Supplementary figures and images for: Transcriptomic analysis reveals the regulatory mechanisms of messenger RNA (mRNA) and long non-coding RNA (lncRNA) in response to waterlogging stress in rye (Secale cereale L.)
Source: BMC Plant Biol. 2024 Jun 12;24:534. doi: 10.1186/s12870-024-05234-x (PMC11167852; doi:10.1186/s12870-024-05234-x)

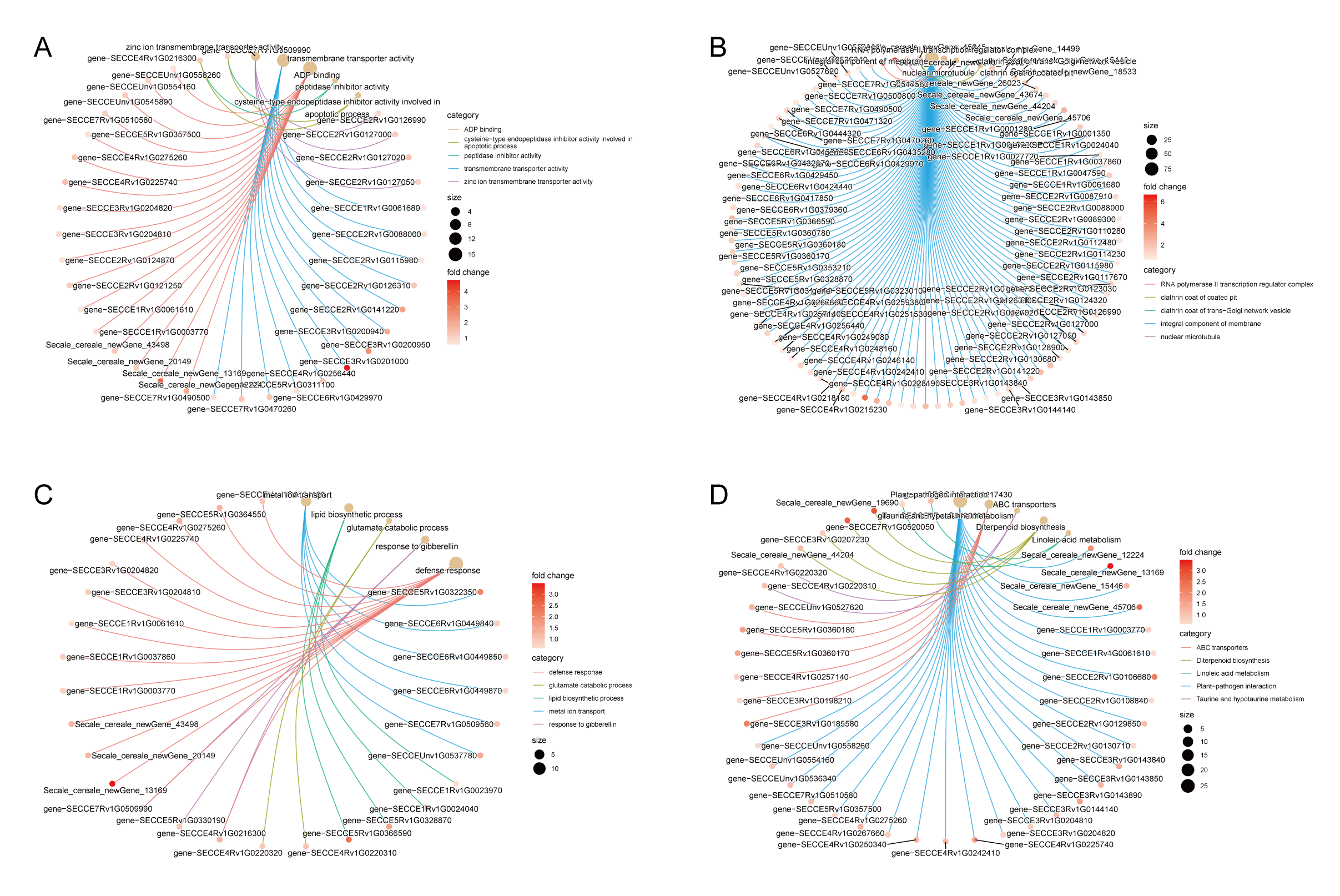

Supplement: Supplementary file 7 — Additional file 7. Enrichment analysis of upregulated DE-mRNAs. Enriched MF (A), enriched CC (B), enriched BP (C), and enriched KEGG pathways (D). [file 12870_2024_5234_MOESM7_ESM.tif]

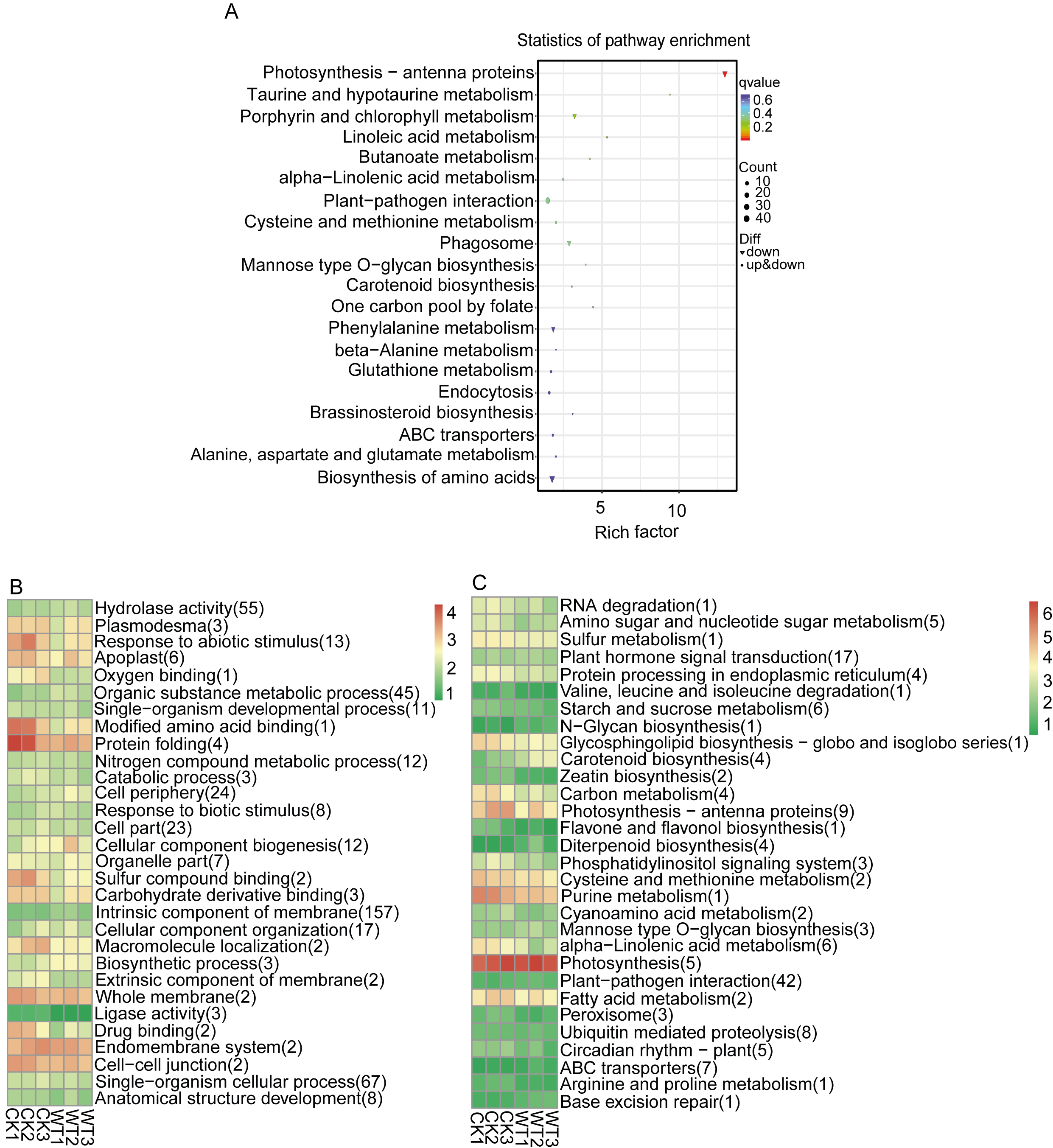

Supplement: Supplementary file 8 — Additional file 8. Protein-protein interaction network of DE-mRNAs [file 12870_2024_5234_MOESM8_ESM.tif]

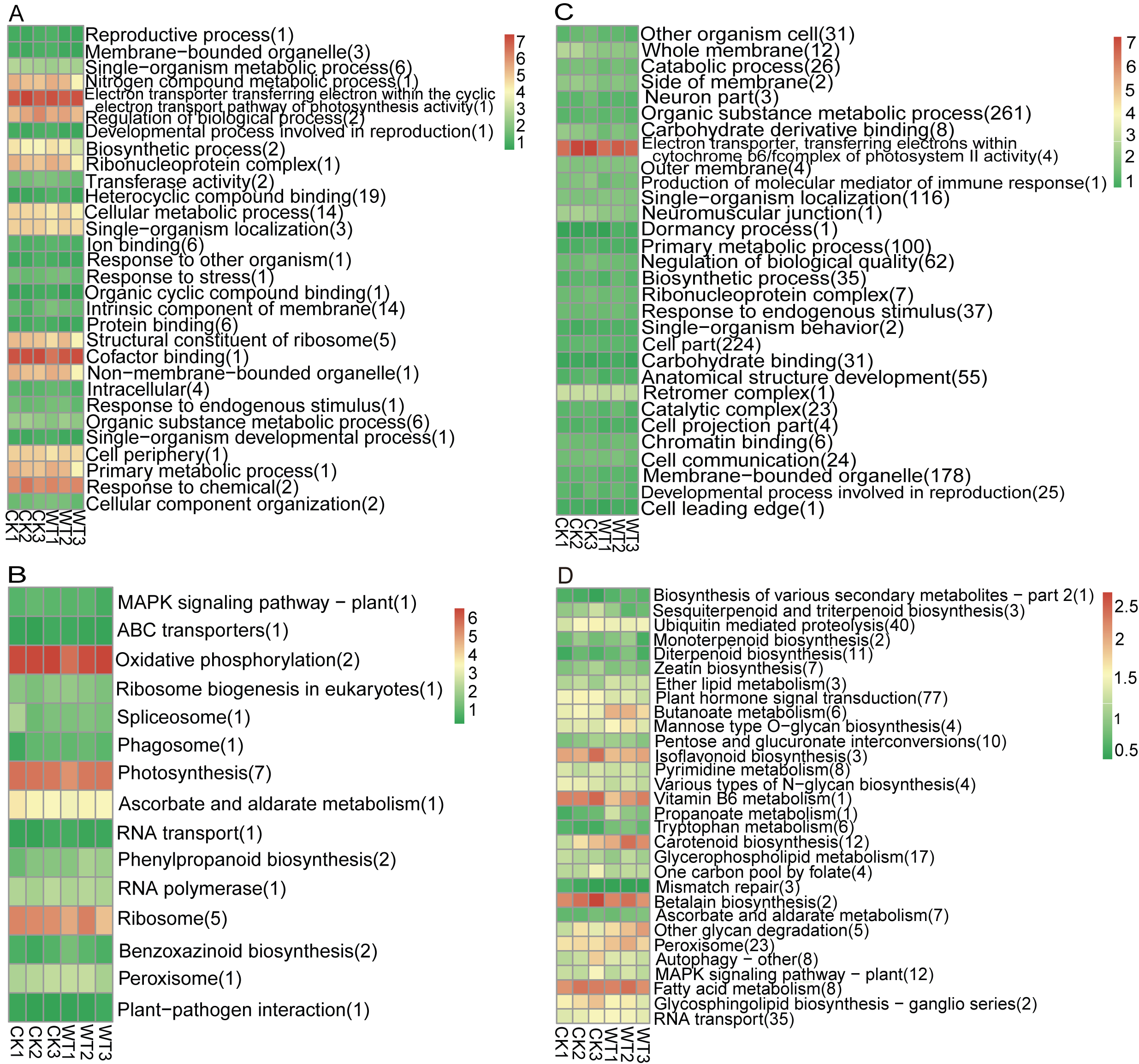

Supplement: Supplementary file 9 — Additional file 9. Enrichment and clustering analysis of DE-mRNAs. Statistics of KEGG pathway enrichment of DE-mRNAs (A), GO term enrichment clustering of DE-mRNA targeted genes (B), and KEGG pathway enrichment clustering of DE-mRNA targeted genes (C). The terms and pathways with higher gene expression levels are shown in red, while those with lower levels are shown in blue; the number of enriched genes associated with each term and pathway is indicated in brackets. [file 12870_2024_5234_MOESM9_ESM.tif]

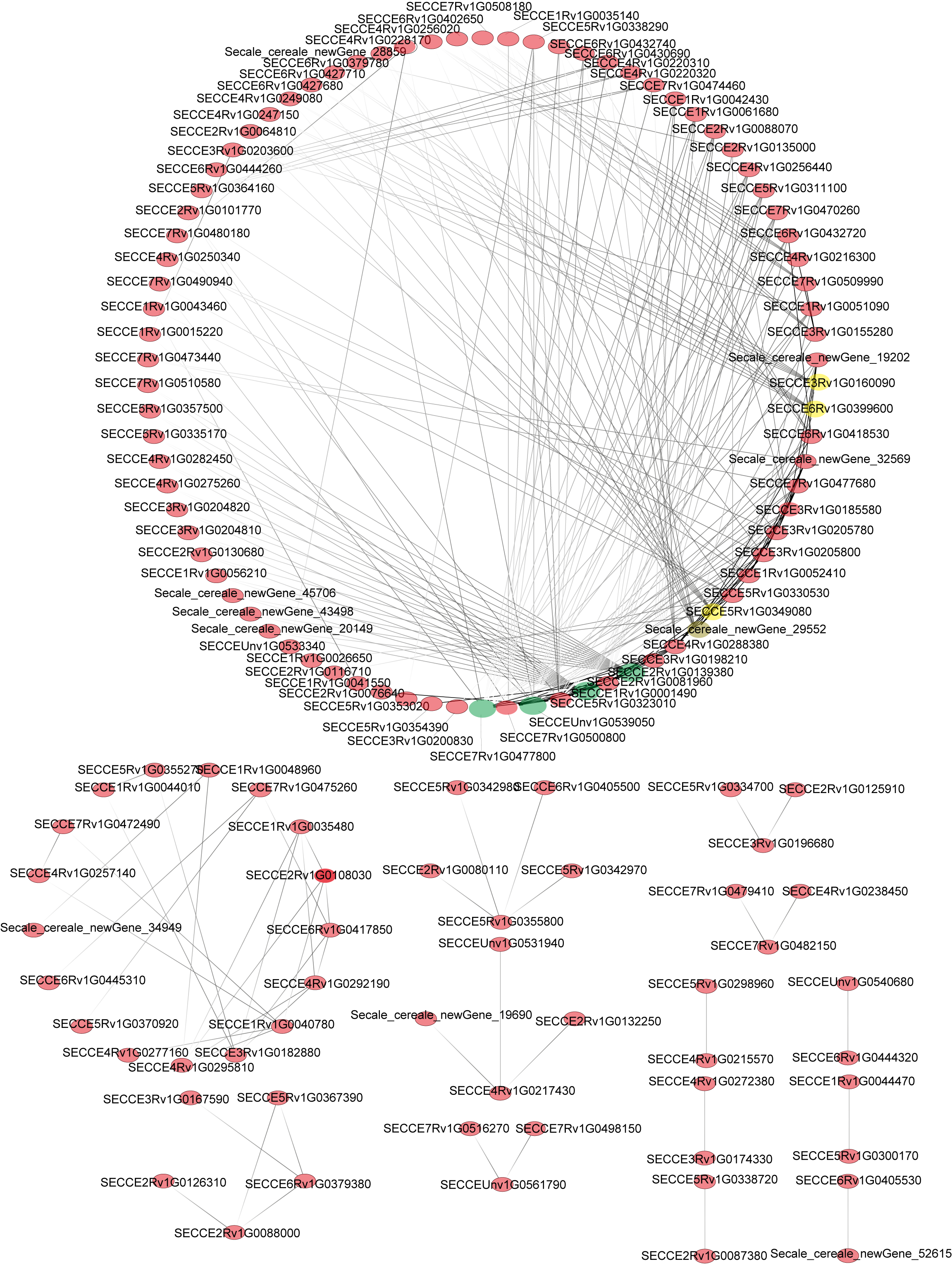

Supplement: Supplementary file 10 — Additional file 10. Enrichment analysis of DE-lncRNA target genes. GO-term enrichment clustering of DE-lncRNA cis-target genes (A), KEGG pathway enrichment clustering of DE-lncRNA cis-target genes (B), GO-term enrichment clustering of DE-lncRNA trans-target genes (C), and KEGG pathway enrichment clustering of DE-lncRNA trans-target genes (D). [file 12870_2024_5234_MOESM10_ESM.tif]
